# Excess Ascorbate is a Chemical Stress Agent against Proteins and Cells

**DOI:** 10.3390/ph13060107

**Published:** 2020-05-27

**Authors:** Maria Lehene, Eva Fischer-Fodor, Florina Scurtu, Niculina D. Hădade, Emese Gal, Augustin C. Mot, Alina Matei, Radu Silaghi-Dumitrescu

**Affiliations:** 1 Department of Chemistry, Babes-Bolyai University, Cluj-Napoca 400028, Romania; mlehene@chem.ubbcluj.ro (M.L.); nbogdan@chem.ubbcluj.ro (N.D.H.); emese@chem.ubbcluj.ro (E.G.); augustinmot@chem.ubbcluj.ro (A.C.M.); mateialina@chem.ubbcluj.ro (A.M.); 2 Tumor Biology Laboratory, Institute of Oncology I. Chiricuta, Cluj-Napoca 400015, Romania; fischer.eva@iocn.ro; 3 Medfuture Research Center for Advanced Medicine, Iuliu Hatieganu University of Medicine and Pharmacy, Cluj-Napoca 400037, Romania; florinadeac@chem.ubbcluj.ro

**Keywords:** hemoglobin, albumin, ascorbate, erythrocyte, antioxidant, leukocyte, COVID-19

## Abstract

Excess ascorbate (as expected in intravenous treatment proposed for COVID-19 management, for example) oxidizes and/or degrades hemoglobin and albumin, as evidenced by UV-vis spectroscopy, gel electrophoresis, and mass spectrometry. It also degrades hemoglobin in intact blood or in isolated erythrocytes. The survival rates and metabolic activities of several leukocyte subsets implicated in the antiviral cellular immune response are also affected. Excess ascorbate is thus an unselective biological stress agent.

## 1. Introduction

Antioxidants, and ascorbate in particular, have long been a subject of ambitious (and at times unsupported) proposed medical treatments [1]. More recently, clinical trials were proposed for intravenous injections of ascorbate against SARS-CoV-2/COVID-19 [2,3,4].

At physiological concentrations (e.g., 50–150 µM in blood) ascorbate is a frontline defense molecule against oxidative stress, with hemoglobin (Hb) being a key beneficiary, and is also an essential contributor to other molecular mechanisms, such as those related to transition-metal-dependent hydroxylases [5,6,7,8,9,10,11,12,13,14,15,16,17,18,19,20,21,22]. However, at much larger concentrations (1 mM and beyond, which is a range occasionally tested in intravenous administration [2]), ascorbate was shown to accelerate the autooxidation of hemoglobin [21]. Other antioxidants, notably including isoascorbate/erythorbate (a stereoisomer of ascorbate), similarly exhibited dual properties as reducing agents at low concentrations and as prooxidants when in excess [13,23,24,25,26,27,28,29,30,31,32]. In fact, ascorbate and isoascorbate were shown to even degrade viruses in vitro, most likely via Fenton chemistry under aerobic conditions; [33] indeed, the Fenton system (arguably the most commonly known example of an oxidative stress model) consists of ascorbate under aerobic conditions, with traces of Fe catalyst [34]. The mechanisms whereby this oxidation occurs were proposed to entail comproportionation reactions of antioxidants; in the case of ascorbate, comproportionation with dehydroascorbate leads to the relatively long-lived (i.e., directly observable by electron paramagnetic spectroscopy) ascorbyl radical [5,28]. Regarding hemoglobin, recent data showed that SARS-CoV-2 produces a protein that attacks hemoglobin directly and extracts the heme, demonstrating potential to generate oxidative stress [35]—perhaps relevant for other manifestations [36,37].

## 2. Results and Discussion

**Figure 1:**
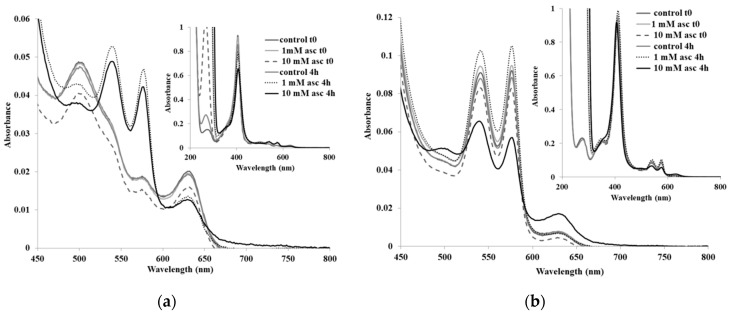
UV-vis spectra of hemoglobin (**a**) panel: ferric; (**b**) panel: ferrous oxy with varying concentrations of ascorbate. Conditions: 5 µM met-Hb and 7 µM oxy-bovine-Hb, room temperature, 50 mM phosphate, pH 7.

Ascorbate degrades not only oxyhemoglobin, but also methemoglobin. Thus, Figure 1 shows the UV-vis spectra of ferric hemoglobin exposed to various concentrations of ascorbate. The initial spectra (immediately after mixing) were essentially identical for all three samples (0, 1, and 10 mM ascorbate). After incubation at room temperature, the control (0 mM ascorbate) sample remained expectedly unchanged. Also, the 1 mM sample developed distinct maxima at 540 and 580 nm, characteristic of ferrous oxyhemoglobin; this was also accompanied by the expected slight decrease in Soret absorption (extinction coefficients at the Soret band: ~150,000 vs. ~120,000 cm^−1^M^−1^), typical of reducing agents [38]. At four hours, the 10 mM sample also displayed distinct features at 540 and 580 nm, typical of the oxy form, but unexpectedly slightly lower than the 1 mM sample, despite the 10-fold increase in reducing agent. The Soret band in the 10 mM sample was also lower than that of the 1 mM sample. The slight broadening of the Soret band in the 10 mM sample suggested that other forms of the heme may have appeared in the solution. Deoxy Hb is a possible candidate, as previously observed at high reductant concentrations [21], implying that the O_2_ in the cuvette was depleted by the ascorbate. The latter may have involved a direct reaction of the ascorbyl radicals and dehydroascorbic acid with molecular oxygen, which, considering that neither of the two reducing agents was a four-electron deliverer, was bound to generate oxidative stress species. More importantly, the 10 mM sample also developed a monotonously-increasing absorbance from 800 to 650 nm, a distinct feature that occurs during partial heme degradation via oxidative reactions with hemoglobin both in vitro and in vivo [20].

The spectra of oxy Hb with ascorbate are perhaps even more relevant; they indicated a distinctly stronger effect (Figure 1). Thus, at four hours, the 10 mM sample, but not the 1 mM, sample halved its oxy maxima of 540 and 580 nm and developed a strong peak at 630 nm, characteristic of the ferric form. Excess ascorbate was thus shown to be an efficient promoter of Hb autooxidation. We previously showed that at longer incubation times and/or with higher ascorbate concentrations, oxy-Hb also developed spectral features of heme degradation, not only oxidation of the iron [21].

**Figure 2:**
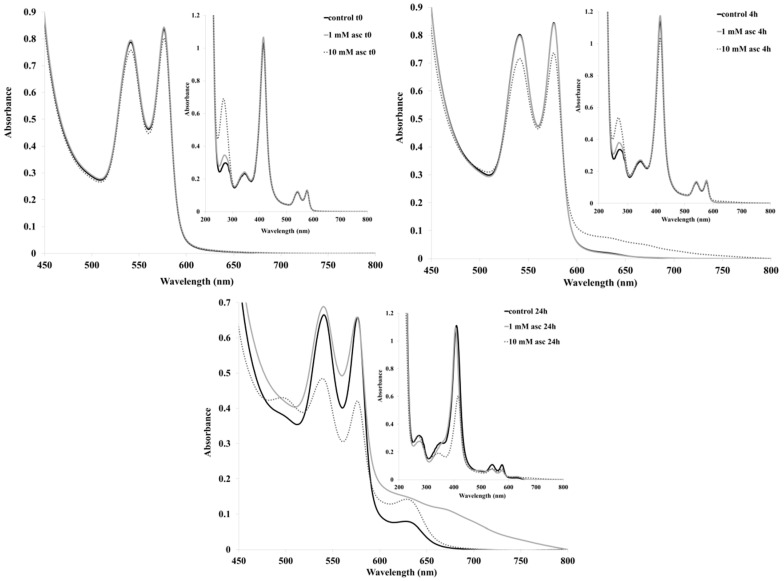
UV-vis spectra of erythrocytes exposed to ascorbate. Conditions: Bovine erythrocytes re-suspended in phosphate buffer saline (pH 7.4, PBS), were incubated at 37 °C. Aliquots of the samples were retrieved at indicated times and diluted with PBS in the UV-vis cuvettes for spectral measurements. Experiments were performed in triplicate.

**Figure 3:**
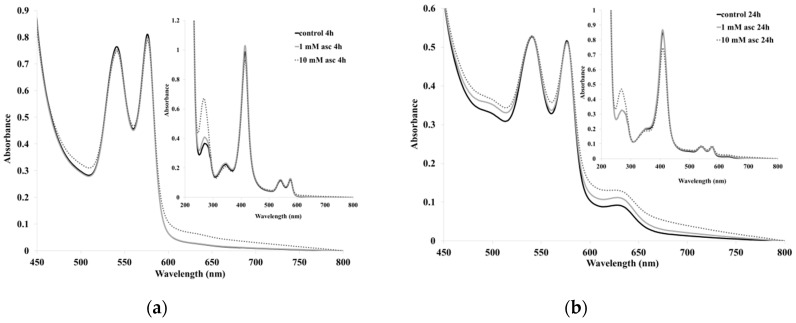
UV-vis spectra of whole blood exposed to ascorbate at (**a**) 4 h and (**b**) 24 h after mixing. Conditions: Bovine blood was incubated at 37 °C. Aliquots of the samples were retrieved at indicated times and diluted with PBS in the UV-vis cuvettes for spectral measurements. Experiments were performed in triplicate.

Figure 2 shows the spectra of whole blood as well as of erythrocytes exposed to two concentrations of ascorbate. Immediately after mixing, a slight decrease in Hb absorption was seen for the 10 mM sample. At four hours, a distinct wide absorbance at 600–800 nm developed in the 10 mM sample, symptomatic of heme degradation. In agreement with this observation, the spectral features of oxy-Hb were distinctly lower at this point than the control and the 1 mM sample. Notably, the ascorbate maximum at ~270 nm also showed a distinct decrease after four hours. At 24 h, the 10 mM sample showed a Soret peak that was reduced to ~50% of its original intensity with a distinct maximum at 630 nm, indicating a large methemoglobin contribution. Importantly, the 1 mM sample also displayed degraded heme features in the 600–800 nm region at this point, while the ascorbate in both samples appeared to be completely consumed. Similar (though milder) observations were made regarding the intact blood exposed to ascorbate (Figure 3). In this case, a Soret band decrease was seen for the 10 mM sample only at 24 h, while the 600−800 nm increases were smaller but still detectable in the 10 mM sample, even at four hours. Plasma antioxidants, such as urate and albumin [7,15,21,39], are indeed expected to additionally protect erythrocytes from stress induced by excess ascorbate.

**Figure 4:**
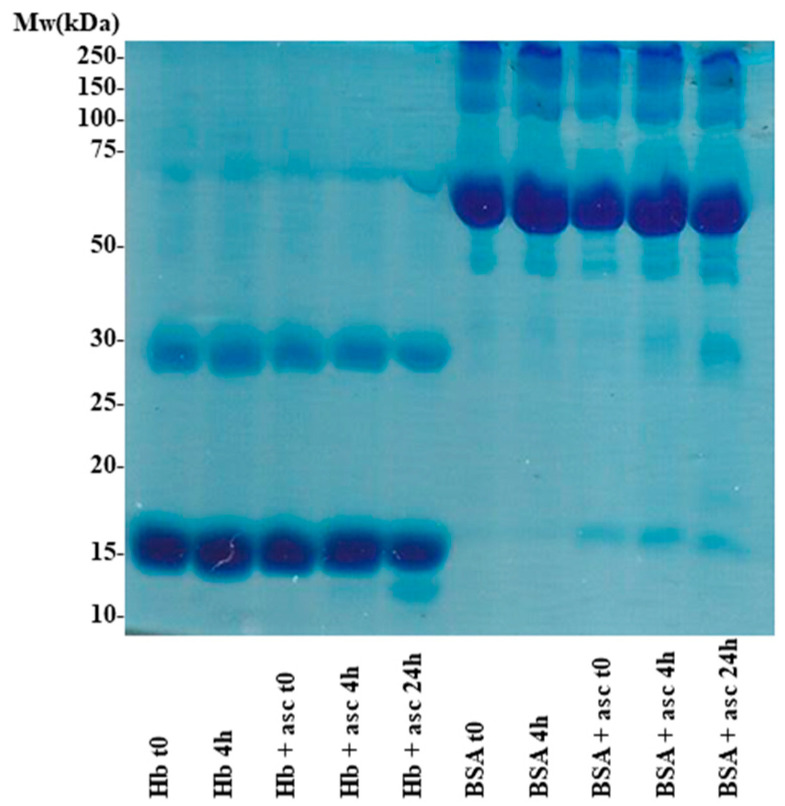
SDS-PAGE (12%) of hemoglobin and albumin exposed to ascorbate. Conditions: 50 µM Hb and 20 µM bovine serum albumin (BSA) were incubated at room temperature for the indicated times with or without 10 mM ascorbate in 50 mM phosphate, pH 7. The samples from left to right are: 1. Hb t0 (no incubation); 2. Hb 4 h; 3.Hb + ascorbate t0; 4. Hb + ascorbate 4 h; 5. Hb + ascorbate 24 h; 6.BSA t0; 7.BSA 4 h; 8.BSA + ascorbate t0; 9.BSA + ascorbate 4 h; 10.BSA + ascorbate 24 h.

Figure 4 shows SDS-PAGE data on Hb exposed to ascorbate; albumin was also employed as an alternative example of a blood protein that may be exposed to excess ascorbate. For the Hb–ascorbate sample, a slight decrease in the intensity of the bands due to the Hb (dominant monomer and less intense dimer) is seen at four hours, alongside the appearance of a new band at a lower molecular weight. For albumin, lower molecular weight bands also developed over time. These findings suggested that a minor part of the protein may be degraded at these ascorbate concentrations, and that this process is not unique to hemoglobin. A direct reaction of ascorbate/ascorbyl with molecular oxygen, or of ascorbyl with proteins, may be responsible for initiating such processes. Attempts were made to verify this hypothesis using mass spectrometry. As shown in Figure S1, major features of the mass spectra were not affected by ascorbate in either of the two proteins. However, in the case of Hb, a clear decrease in the intensity of the β subunits was observed compared to the α subunit after treatment with ascorbate; also, new distinct peaks with peptide-like mass were detectable, in agreement with the SDS-PAGE findings. A similar observation was previously noted when examining the behavior of Hb with other oxidative stress agents [40]. For albumin, due to its high heterogeneity and lower purity, only minor changes in the region corresponding to small peptides were identified in the mass spectra of ascorbate-treated samples (Figure S2). Further exploration of these spectra, including purification of the small peptides apparent in the SDS-PAGE and subsequent spectral analyses, may offer better mechanistic insight.

**Figure 5:**
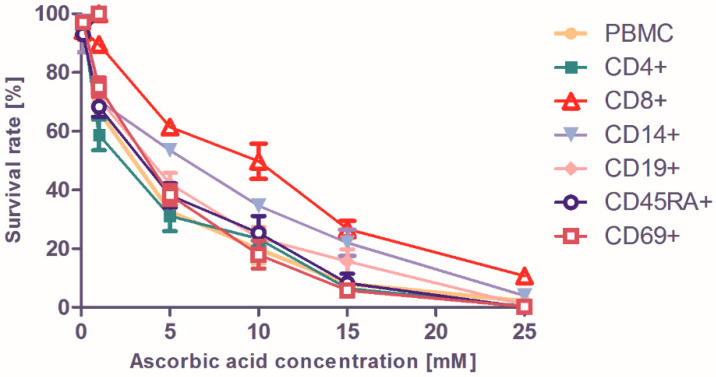
Survival rates for human cell cultures exposed to indicated concentrations of ascorbate for 24 h.

**Figure 6:**
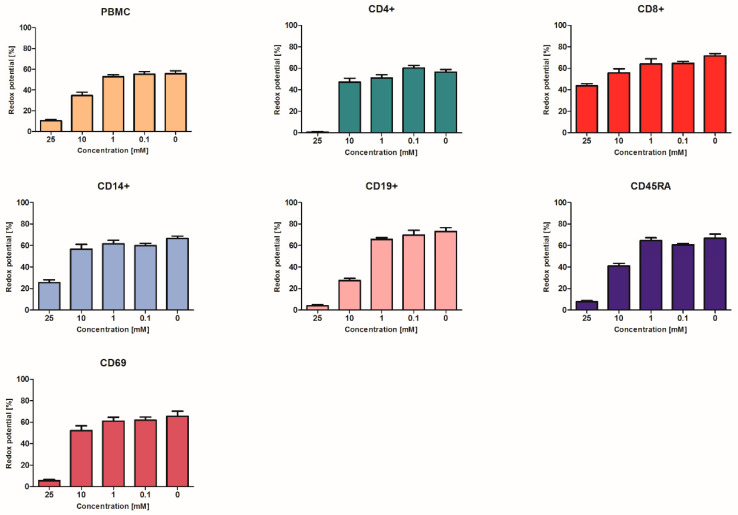
Peripheral blood mononuclear cells (PBMC) and PBMC subsets, i.e., CD4+, CD8+, CD14+, CD19+, CD45RA+, and CD69+, metabolic activities expressed as reduction rates of resazurin dye. For all cell groups, ANOVA *p* values < 0.000, except the CD8+ subset, for which *p* < 0.005. Using the t-test for independent samples, the lowest ascorbic acid concentrations that significantly differed from the control was 10 mM for PMBC (*p* < 0.01), 25 mM for CD4+ (*p* < 0.000), 10 mM for CD8+ (*p* < 0.05), 25 mM for CD14+ (*p* < 0.001), 10 mM for CD19+ (*p* < 0.001), 10 mM for CD45RA+ (*p* < 0.005), and 25 mM for CD69+ (*p* < 0.001).

To verify whether the above-discussed erythrocyte behavior was also mirrored by other types of cells, a number of other (cultured) human cells were also exposed to ascorbate (Figure 5 and Figure 6). The antiviral immune response involves innate mechanisms driven by peripheral blood mononuclear cell (PBMC) subsets, cytotoxic (CD8-positive) and helper (CD4-positive) T cells, B cells characterized by the expression of CD19+ epitopes, and CD14-positive monocytes [41]; the roles of these subpopulations were previously highlighted in COVID-19 infection [42,43]. The changes in CD45RA-expressing naïve T cells proportions, alongside the aberrant activation of helper cells via CD69 upregulation in COVID-19, are important facets of immune dysregulation [43,44]. Examining these PBMC subsets, a notable dose-dependent effect was seen for all types of cells, with relatively similar behaviors showing decreases in viability. For almost all cell types, effects were seen at 1 mM, while the survival rates at 10 mM were almost halved (Figure 5). In regard to metabolic rates, the effects were visible at higher concentrations (Figure 6), with the least affected being the CD8+ killer T cells and the monocytes being mildly affected.

## 3. Materials and Methods

Bovine hemoglobin was obtained as previously described [45]. Proteins were manipulated in 137 mM NaCl, 2.7 mM KCl, and 12 mM NaH_2_PO_4_, pH 7.4 (phosphate buffer saline, PBS) water, or in 50 mM ammonium acetate buffer, as indicated; their concentrations are given per monomer. Bovine serum albumin (BSA fraction V, Sigma, Germany) was used as provided without further purification.

The UV-vis spectra were recorded on Cary 50 (Agilent, Inc.,Santa Clara, CA, USA) and Lambda 25 (PerkinElmer, Waltham, MA, USA) instruments. For mass spectra, the protein solution was injected directly into an LTQ-Orbitrap XL spectrometer (Thermo Fisher Scientific, Waltham MA, USA); the ionization mode was ESI (electrospray, positive mode) and the mass spectrum (MS) conditions were as follows: vaporization temperature 250 °C, sheath gas (nitrogen) flow rate 20 (a.u., arbitrary units), auxiliary gas (nitrogen) flow rate 10 (a.u.), sweep gas (nitrogen) flow 5 (a.u.) source voltage 6 kV, capillary temperature 275 °C, capillary voltage 30 V, and tube lens 245 V.

Blood collection was conducted according to the approval of the Ethics Committee and Animal Protection for experiments from the Institute of Biological Research, NIRDBS branch, Cluj-Napoca, Romania (decision 1/28.02.2013).

For in vitro testing on peripheral blood mononuclear cells (PBMC), the cells were harvested previously under the frame of phase V (2016) PN-II-ID-PCE-2011-3-1057 project activities. A healthy male volunteer, aged 44, donated the peripheral blood following provision of written informed consent. The lymphocytes were isolated on Histopaque 1.077 cell separation solution (from Sigma Aldrich St. Louis, MO, USA), following protocol described elsewhere [46], aliquoted, and kept in a liquid nitrogen tank (Cryosystem 2000, MVE Bio-Medical Division, Burnsville, USA)

From the whole PBMC population, the CD4+, CD8+, CD14+, CD19+, CD45RA+, and CD69+ subsets were isolated using magnetic separation methods [46] (MACS system, MS magnetic columns, and magnetic beads from MiltenyiBiotec, BergischGladbach, Germany). The whole PBMC population was placed on 24-well plates with a cell suspension density of 10^5^ cells/mL of cell culture media (RPMI-1640 supplemented with 10% fetal calf serum, 1% nonessential amino acid solution, 1% sodium pyruvate, and 1% glutamine solution; all media and supplements were obtained from Sigma Aldrich). On each plate, 15 distinct wells were filled with cells from the same subpopulation and cell culture media was dispensed into 3 wells without cells. After 24 h in an incubator (Galaxy 48R Brunswick, Eppendorf GmbH, Vienna, Austria), the wells were treated in triplicate with ascorbic acid to obtain final concentrations of 25 mM, 10 mM, 1 mM, or 0.1 mM ascorbic acid in cell culture media. After 24 h of exposure, the cells were removed from the plates, washed twice with phosphate buffer saline solution (PBS, Sigma Aldrich), and resuspended in 200 µL of fresh cell culture media. Every sample was then divided in two parts of 100 µL each and loaded on two different 96-well black microplates with transparent bottoms (Greiner BioOne GmbH, Frickenhausen, Germany), alongside 3 wells containing culture media only as a reference.

In the first plate, every well was treated with 10 µL Alamar Blue cell viability stain (Invitrogen, Thermo Fisher Scientific, Waltham, MA, USA) as described previously [47]. After 2 h of incubation the plates were measured at 540 nm/620 nm emission using a Synergy2 multiplate reader (BioTek Company, Winooski, VT, USA). The fluorescence intensity values for each sample, provided by Gen5 software, were processed to obtain the percentage of reduction of the Alamar Blue dye.

The cell viability of lymphocytes was assessed as described earlier [46]. In the second assay plate loaded with samples, the wells were treated with 20 µL of [3-(4,5-dimethylthiazol-2-yl)-5-(3-carboxymethoxyphenyl)] 2-(4-sulfophenyl)-2H-tetrazolium salt and phenazinemethosulfate mixture (MTS:PMS in a volume proportion of 20:1), with the reagents comprising the CellTiter 96 Aqueous Non-Radioactive Cell Proliferation Assay kit from Promega Corporation, Madison, WI, USA. The plates were incubated for 3 h and absorbance was measured at 492 nm with the above-mentioned Synergy2 equipment. We used the cell culture medium as a color control and untreated cells as a 100% viability reference. The MTS color intensity was directly proportional with the number of living cells in the treated samples (in triplicates), therefore, the median survival ratio was calculated.

Statistical analysis included one-way ANOVA to test the null hypothesis of multiple means equality and Student’s t-test for independent samples to test the null hypothesis of two-means nondifference, with *p* = 0.05 as the statistical threshold for significance. Statistica software (StatSoft Inc., Tulsa, OK, USA) was employed for the statistical analysis.

## 4. Conclusions

In large concentrations, ascorbate individually affects/alters proteins by mechanisms involving oxidative stress and entailing modification/degradation of the polypeptide, besides redox changes at metal sites such as hemoglobin. These effects are minor, but also visible in whole red blood cells and indeed in whole blood in vitro. Cells of the immune system are also affected by excess ascorbate, suggesting that oxidative stress-related molecular mechanisms exist and may be triggered in experiments/treatments where excess ascorbate is injected intravenously. Clearly these mechanisms (and the levels of chemical stress entailed with respect to proteins) are sensitive to ascorbate concentrations, to the extent that ascorbate returns to its physiologically innocuous role of a mild reductant at lower concentrations. The strong effects on erythrocytes and leukocytes, as well as the simple oxidative nature of the processes generated by ascorbyl radicals, suggest that ascorbate-induced oxidative damage may well affect other classes of biomolecules besides proteins.

The physiological concentrations of ascorbate in blood are maintained well below 1 mM, the limit at which the present study detected stress effects on erythrocytes and on PMBC cell lines. It must be clearly stated that, under these conditions, dietary supplementation with ascorbate is unlikely to have notable physiological effects on blood or on antiviral responses of the organism, although analogous effects on the microbiome within the digestive system are expected with those on human cells shown here, and may indirectly impact the rest of the organism either positively or negatively. Therefore, this area may benefit from further detailed exploration.

Previous studies showed that viruses may be inactivated by ascorbate and other antioxidants via Fenton-type mechanisms in vitro [33]. Before considering ascorbate as a treatment for COVID-19, it must be stressed that (1) such actions were only demonstrated in vitro and (2) in the present study, normal human cells were also shown to be affected at high ascorbate concentrations. None of these data support a hypothesis where SARS-CoV-2 could be inactivated in vivo by intravenous ascorbate. Recent studies [3,4] described that 2–10 g/day vitamin C doses were given to COVID-19 patients, and this therapy improved patients’ oxygenation indexes; however, no experimental data were given and the only reference was a nonpeer-reviewed web post. Also, the phase II clinical trial (US National Library of Medicine https://clinicaltrials.gov/ct2/show/NCT04264533) cited by several papers has not been updated since early March 2020, with no results were released until the present time.

Hence, intravenous ascorbate cannot be proposed as a cure for COVID-19. On the other hand, the clear effect of mM ascorbate on immune cells raises the question of whether intravenous ascorbate (or indeed other compounds capable of similar action, such as isoascorbate) may afford a way to mitigate cytokine overexpression (cytokine storm) [44] by such cells under the effect of COVID-19. The main player in this cytokine storm, incriminated in COVID-19 as well, is interleukin-6 (IL-6), which is secreted by innate immune cells and monocytes and is also a fine-tuning regulator of CD4+, CD8+ T cells, and B cells and activates monocytes [41]. Since excess ascorbate causes a serious imbalance in these PBMC subsets, integrity of the immune system may be threatened even more strongly. On the other hand, as circulating ascorbate levels fall, its antioxidant properties may become more important. This may be relevant when one considers that oxidative stress (involving heme release and hence degradation of erythrocytes) is in fact one of the side-effects of COVID-19 [35]. In this context, it should be noted that oxidative stress due to uncontrolled iron management (such as related to heme) is managed, among others, by iron chelators, some of which also have the ability not only to chelate iron but also to quench free radicals generated by free iron, e.g., hydroxamate-based chelators or ethylene diaminotetraacetic acid (EDTA) [24,32]. The latter is arguably also relevant because it additionally displays anticoagulant properties, which may be relevant given the observed thrombosis episodes in COVID-19 [36,37].

## Supplementary Materials

Can be found at https://www.mdpi.com/1424-8247/13/6/107/s1, Figure S1: ESI(+)MS spectra of met hemoglobin before and after ascorbate treatment, Figure S2: ESI(+)MS spectra of albumin before and after ascorbate treatment.
