# Supplementary material for: Excess Ascorbate is a Chemical Stress Agent against Proteins and Cells
Source: Pharmaceuticals (Basel). 2020 May 27;13(6):107. doi: 10.3390/ph13060107 (PMC7344896; doi:10.3390/ph13060107)
Supplement: Supplementary file 1 [file pharmaceuticals-13-00107-s001.pdf]

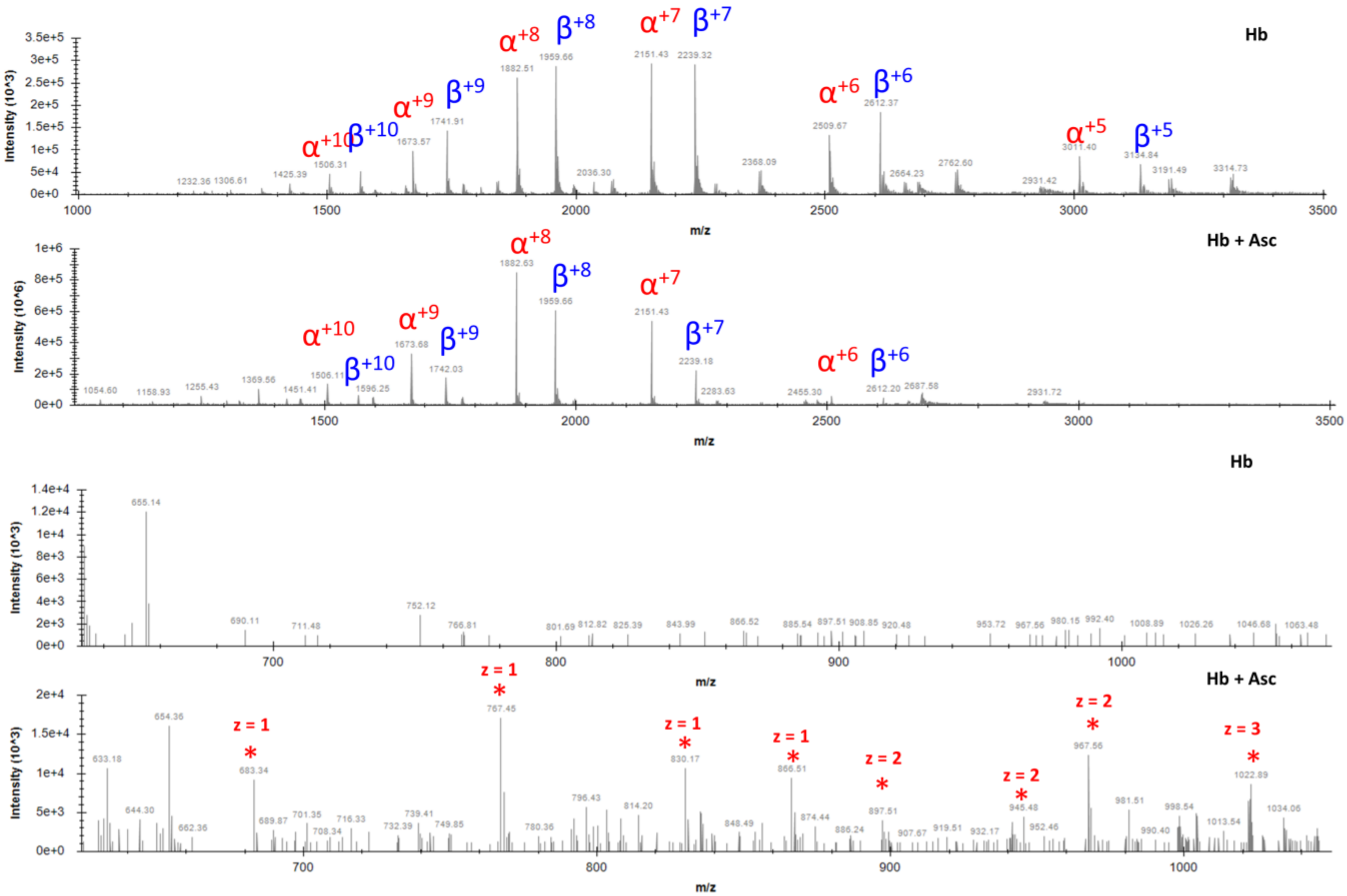

Figure S1. ESI(+)MS spectra of met hemoglobin before and after ascorbate treatment (in ammonium acetate buffer, pH 5), in the whole m/z region as well as in the low m/z region.The α and β subunits are indicated along with the corresponding charge. The extra peaks in the low m/z region are indicated with * and their charge.

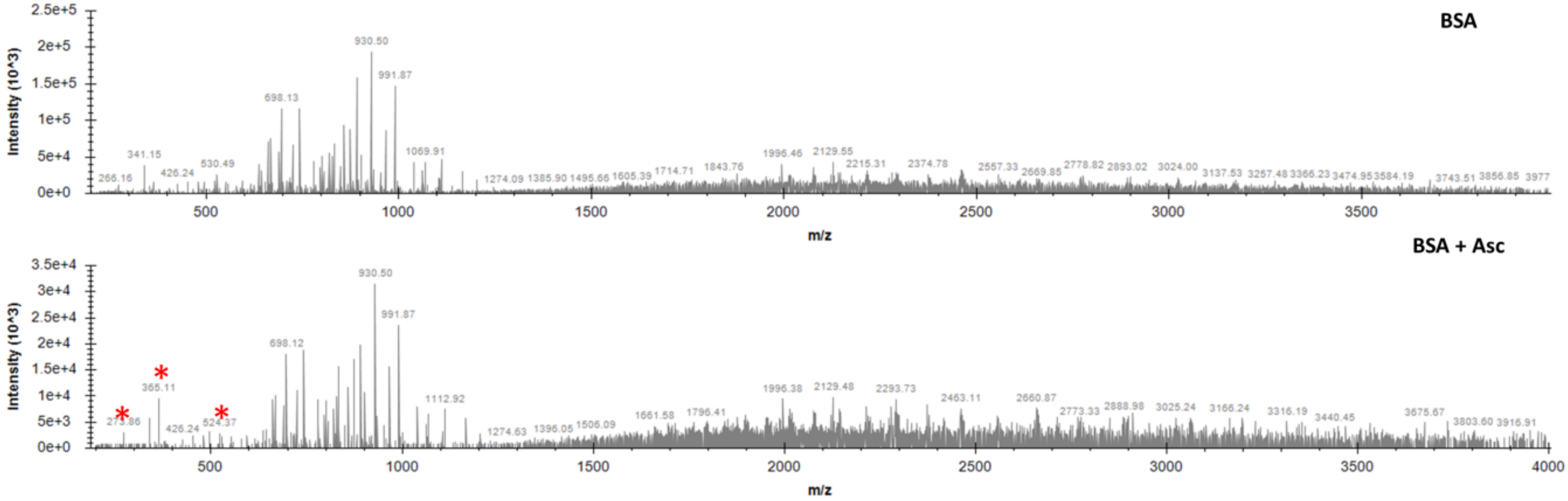

Figure S2. ESI(+)MS spectra of albumin before and after ascorbate treatment (in ammonium acetate buffer, pH 5). The extra peaks in the low m/z region are indicated with * and their charge.
